# Magnetometer and Gyroscope Calibration Method with Level Rotation

**DOI:** 10.3390/s18030748

**Published:** 2018-03-01

**Authors:** Zongkai Wu, Wei Wang

**Affiliations:** College of Automation, Harbin Engineering University, No. 145 Nantong Street, Harbin 150001, China; wuzongkai407@hrbeu.edu.cn

**Keywords:** level rotation, cubature Kalman filter (CKF), magnetometer and gyroscope calibration

## Abstract

Micro electro mechanical system (MEMS) gyroscopes and magnetometers are usually integrated into a sensor module or chip and widely used in a variety of applications. In existing integrated gyroscope and magnetometer calibration methods, rotation in all possible orientations is a necessary condition for a good calibration result. However, rotation around two or more axes is difficult to attain, as it is limited by the range of movement of vehicles such as cars, ships, or planes. To solve this problem, this paper proposes an integrated magnetometer and gyroscope calibration method with level rotation. The proposed method presents a redefined magnetometer output model using level attitude. New gyroscope and magnetometer calibration models are then deduced. In addition, a simplified cubature Kalman filter (CKF) is established to estimate calibration parameters. This method possesses important value for application in actual systems, as it only needs level rotation for real-time calibration of gyroscopes and magnetometers. Theoretical analysis and test results verify the validity and feasibility of this method.

## 1. Introduction

Micro-electro-mechanical-system inertial measurement units (MEMS-IMUs) commonly include a gyroscope, accelerometer, and magnetometer, and are widely employed in many fields which rely on their small size and low costs. The gyroscope can sense the vehicle angular velocity and calculate the attitude change. The magnetometer and accelerometer measure the local magnetic field and the acceleration of the rigidly-attached platform. However, the precision of MEMS gyroscopes is generally not high and attitude error may accumulate quickly. Therefore, MEMS gyroscopes must be calibrated before use. In general, gyroscope calibration methods need to be supplemented by external information. One of the common methods is based on Global Navigation Satellite System (GNSS)-aided calibration [1,2,3]. In dynamic environments, with the use of the precise positioning and velocity information, a GNSS- or GNSS/MEMS-IMU-integrated navigation system can estimate gyroscope bias in real-time and obtain accurate attitude. However, it is impossible to achieve good performance in a static environment. Furthermore, GNSS signals are not always available on account of occlusions, such as buildings, viaducts, tunnels, and dense forests. To improve the reliability and accuracy of the navigation solution, a magnetometer has been introduced to calibrate the gyroscope.

A magnetometer must be calibrated before use because it is prone to being influenced by electromagnetic disturbance. Many magnetometer calibration methods have been presented in related literature. Ellipsoid fitting is a common technology to calibrate a magnetometer [4,5]. This technology does not need any external information because it fits an ellipsoid locus from a non-calibrated magnetometer to a sphere. The advantages of this technology are ease of use, short run-time, and reduced computational needs. However, it also has the weakness of low precision and poor adaptability. Magnetometers can also be calibrated using external attitude information—especially heading information [6,7]. A common drawback of these methods is that erroneous attitude information may lead to worse calibration results. “Attitude-independent” is a classic magnetometer calibration method [8,9,10,11,12,13,14]. By removing the attitude matrix, this method obtains a model that does not contain any attitude information, and a nonlinear Kalman filter (KF) is used to estimate calibration parameters. Many methods have been presented based on this model. For example, attitude-independent magnetometer calibration with time-varying bias was proposed in Reference [11]. Many types of algorithms have been presented to improve the calibration accuracy and adaptability in nonlinear systems, such as extended Kalman filter (EKF) [12], neural network [13], particle filter (PF), and so on [14]. The EKF approach adapts techniques from calculus—namely multivariate Taylor series expansions—to linearize a model near a working approximation point. If the system model (as described below) is not well-known or is inaccurate, then Monte Carlo methods—especially particle filters—are employed for estimation [15,16,17]. For a sensor’s static bias (additive error) and to represent sensitivity error, a neural network method is used to solve the problem. However, it is difficult to set appropriate initial parameters in PF, neural network, or EKF approaches. A particle swarm optimization algorithm is used to calibrate the magnetometer, as it depends on a more accurate nonlinear model and need not consider the initial estimation parameters [18,19,20]. However, this algorithm has high computational cost, which is difficult to implement. Recently, integrated calibration methods for gyroscopes and magnetometers have been widely developed in the literature [21,22]. These methods calibrate a magnetometer using the angular velocity from the gyroscope, while gyroscope calibration parameters are simultaneously estimated using the calibrated magnetometer readings. Through simulation and testing in the field, it is shown that the method is practical, useful, and adaptable to magnetic disturbance. The major shortcoming of this method is the necessity of the initial attitude information. Recently, a virtual rotation scheme was proposed as an advanced calibration method [23,24]. The response of the gyroscope to virtual rotation can be used to calibrate the bias and scale factor errors [25], but this method has not yet been used to calibrate a magnetometer.

The advantages and disadvantages of the above methods are summarized in Table 1. From the above, the precision magnetometer model and accurate external information are important for the calibration result. On the other hand, most methods require that the sensor be rotated in all possible orientations to guarantee a good result. In many navigation systems on vehicles such as cars, ships or planes, most of rotation orientations cannot be performed, which leads to calibration parameters being incorrectly estimated and may have worse performance than non-calibrated systems. To solve the problem, this paper presents a method to calibrate magnetometers and gyroscopes with level rotation, and without heading information. In this method, the three-dimensional model of calibrating the magnetometer is reduced to a two-dimensional model using the level attitude. Based on this idea, the magnetometer output model is redefined, and then a new gyroscope and magnetometer calibration model is proposed, and finally a simplified cubature Kalman filter (CKF) is designed to complete the calibration.

The rest of this paper is organized as follows. Section 2 demonstrates the redefined magnetometer output model and introduces the derivation process of the new calibration model and the filter algorithm. Simulation tests are performed in Section 3. The test result is shown in Section 4 by applying an actual MEMS-IMU system, and conclusions are given in Section 5.

## 2. System Modeling and Filter

In this section, the leveled output model of the magnetometer is introduced by analyzing the characteristics of level rotation. Based on this model, the magnetometer and gyroscope calibration models are constructed. Finally, a simplified CKF is designed to estimate the calibration parameters.

**Figure 1:**
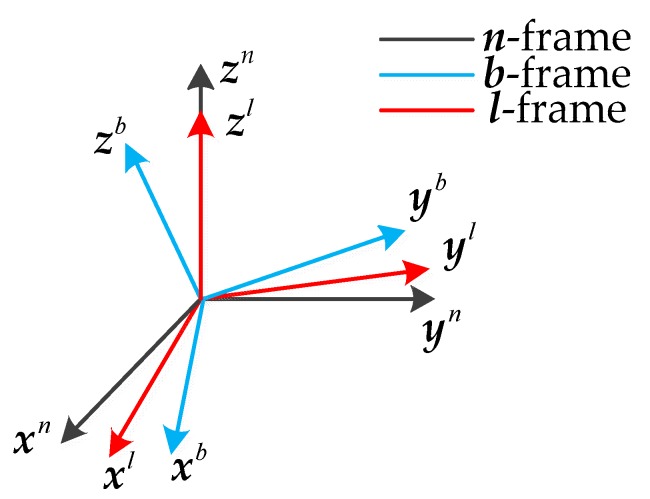
Sketch of the coordinate systems.

The frame definitions are introduced as follows, and a sketch of the coordinate systems is shown in Figure 1.

- Navigation frame (***n***-frame): The navigation frame is located on the vehicle. It points to the east, north, and upward.
- Body frame (***b***-frame): The body frame is fixed to the vehicle. Its *x*-axis and *y*-axis point to the right and forward of the vehicle, and its *z*-axis follows the right-hand rule.
- Leveled frame (***l***-frame): The leveled frame is also located on the vehicle. It is the frame by leveling ***b***-frame such that its *z*-axis is parallel to the upward vertical and possesses an error angle with respect to the ***n***-frame.

### 2.1. Magnetometer Model

The magnetometer measurement model can be expressed as [26]:

(1) $$B_{m}=C_{sc}C_{no}(C_{si}B_{t}+b_{hi})+b_{b}+\varepsilon_{m},$$

where $B_{m}$ is the measurement from the magnetometer and $B_{t}$ is the true magnetic field. $C_{sc}$ is a scale factor matrix, $C_{no}$ is a non-orthogonal matrix and $C_{si}$ is a soft-iron effect matrix. $b_{hi}$ and $b_{b}$ are, respectively, the hard-iron effect vector and bias. $\varepsilon_{m}$ is the measurement noise of the magnetometer, which can be modeled as Gaussian white noise. $C_{sc}$, $C_{no}$, and $C_{si}$ are specifically expressed as follows:

(2) $$C_{sc}=\left[\begin{array}{ccc} C_{scx} & 0 & 0\\ 0 & C_{scy} & 0\\ 0 & 0 & C_{scz} \end{array}\right],$$

(3) $$C_{no}=\left[\begin{array}{ccc} 0 & C_{noxy} & C_{noxz}\\ C_{noyx} & 0 & C_{noyz}\\ C_{nozx} & C_{nozy} & 0 \end{array}\right],$$

(4) $$C_{si}=\left[\begin{array}{ccc} C_{six} & C_{sixy} & C_{sixz}\\ C_{siyx} & C_{siy} & C_{siyz}\\ C_{sizx} & C_{sizy} & C_{siz} \end{array}\right].$$

For the measurement of the magnetometer in the ***b***-frame, Equation (1) can be rewritten as

(5) $$B_{m}^{b}=CB_{t}^{b}+b_{m}^{b}+\varepsilon_{m},$$

in which

(6) $$C=C_{sc}C_{no}C_{si}=\left[\begin{array}{ccc} C_{11} & C_{12} & C_{13}\\ C_{21} & C_{22} & C_{23}\\ C_{31} & C_{32} & C_{33} \end{array}\right],$$

(7) $$b_{m}^{b}=C_{sc}C_{no}b_{hi}+b_{b},$$

and $B_{t}$ is in ***b***-frame such that $B_{t}^{b}=C_{n}^{b}H^{n}$, in which $C_{n}^{b}$ is the ***n***-frame to ***b***-frame rotation matrix and $H^{n}$ is the ***n***-frame geomagnetic field.

When $B_{m}$ is redefined as the leveled magnetometer measurement in ***l***-frame, $B_{t}$ can be changed to equal $C_{n}^{l}H^{n}$, in which $C_{n}^{l}$ is the ***n***-frame to ***l***-frame rotation matrix and can be calculated by the level attitude. Equation (5) can then be rewritten as

(8) $$B_{m}^{l}=CC_{n}^{l}H^{n}+b_{m}^{l}+\varepsilon_{m}.$$

Because the bias in the ***b***-frame is a goal of magnetometer calibration, Equation (8) can be transformed to

(9) $$B_{m}^{l}=CC_{n}^{l}H^{n}+C_{b}^{l}b_{m}^{b}+\varepsilon_{m},$$

in which $C_{b}^{l}$ is the ***b***-frame to ***l***-frame rotation matrix provided by the level attitude. For convenience of the next calculation, C is represented as

(10) $$C={\left(I_{3\times 3}+C^{l}\right)}^{-1},$$

(11) $$B_{m}^{l}={\left(I_{3\times 3}+C^{l}\right)}^{-1}C_{n}^{l}H^{n}+C_{b}^{l}b_{m}^{b}+\varepsilon_{m},$$

where $I_{3\times 3}$ is an identity matrix.

In this paper, as the algorithm is designed for level rotation, this situation may lead to lower-quality observation of some calibration parameters. To avoid the error introduced by unobservable parameters, $C^{l}$ is assumed to be a symmetric matrix [11]:

(12) $$C^{l}=\left[\begin{array}{ccc} C_{11} & C_{12} & C_{13}\\ C_{12} & C_{22} & C_{23}\\ C_{13} & C_{23} & C_{33} \end{array}\right].$$

After the leveled magnetometer model is obtained, the calibration model for unknown parameters C and $b_{m}^{b}$ needs to be established. From the above deduction, it is known that $B_{m}^{l}$ are direct magnetometer readings, $H^{n}$ can be acquired from the International Geomagnetic Reference Field (IGRF) model [27] and the norm of $H^{n}$ satisfies

(13) $$\begin{array}{cc} \|H^{n}{\|}^{2} & ={\left(C_{b}^{n}B_{t}\right)}^{T}\left(C_{b}^{n}B_{t}\right)={\left(B_{t}\right)}^{T}C_{n}^{b}C_{b}^{n}B_{t}=\|B_{t}{\|}^{2}\\ & ={\left(C_{l}^{n}B_{t}^{l}\right)}^{T}\left(C_{l}^{n}B_{t}^{l}\right)={\left(B_{t}^{l}\right)}^{T}C_{n}^{l}C_{l}^{n}B_{t}^{l}=\|B_{t}^{l}{\|}^{2} \end{array}.$$

Hence, the relationship between the known ${\left\|H^{n}\right\|}^{2}$ and calibration parameters can be built. The transformation of Equation (9) can be obtained as:

(14) $$C_{n}^{l}H^{n}=\left(I_{3\times 3}+C^{l}\right)\left(B_{m}^{l}-C_{b}^{l}b_{m}^{b}-\varepsilon_{m}\right).$$

The normalized $H^{n}$ can be expressed by

(15) $$\begin{array}{cc} \|H^{n}{\|}^{2}={\left(C_{n}^{l}H^{n}\right)}^{T}C_{n}^{l}H^{n} & ={\left(\left(I_{3\times 3}+C^{l}\right)\left(B_{m}^{l}-C_{b}^{l}b_{m}^{b}-\varepsilon_{m}\right)\right)}^{T}\left(\left(I_{3\times 3}+C^{l}\right)\left(B_{m}^{l}-C_{b}^{l}b_{m}^{b}-\varepsilon_{m}\right)\right)\\ & ={\left(B_{m}^{l}\right)}^{T}\left(I_{3\times 3}+2C^{l}+{\left(C^{l}\right)}^{2}\right)B_{m}^{l}-2{\left(B_{m}^{l}\right)}^{T}\left(I_{3\times 3}+C^{l}\right)C_{b}^{l}b_{m}^{b}\\ & +\|b_{m}^{b}{\|}^{2}+\|\varepsilon_{m}{\|}^{2}-2{\left[\left(I_{3\times 3}+C^{l}\right)B_{m}^{l}-C_{b}^{l}b_{m}^{b}\right]}^{T}\varepsilon_{m} \end{array}.$$

The measuring equation of the magnetometer calibration model can be given by

(16) $$\begin{array}{c} \|H^{n}{\|}^{2}-\|B_{m}^{l}{\|}^{2}={\left(B_{m}^{l}\right)}^{T}\left(2C^{l}+{\left(C^{l}\right)}^{2}\right)B_{m}^{l}-2{\left(B_{m}^{l}\right)}^{T}\left(I_{3\times 3}+C^{l}\right)C_{b}^{l}b_{m}^{b}+\|b_{m}^{b}{\|}^{2}+w_{1}\\ w_{1}=\|\varepsilon_{m}{\|}^{2}-2{\left[\left(I_{3\times 3}+C^{l}\right)B_{m}^{l}-C_{b}^{l}b_{m}^{b}\right]}^{T}\varepsilon_{m} \end{array}.$$

### 2.2. Gyroscope Model

The common gyroscope output model is given by [21,22]:

(17) $$\omega_{g}^{b}=C_{g}\omega_{t}^{b}+b_{g}^{b}+\varepsilon_{g},$$

(18) $${\dot{b}}_{g}^{b}=\varepsilon_{u},$$

(19) $$C_{g}=C_{gno}C_{gsc}C_{gmis},$$

where $\omega_{g}^{b}$ is the measured value from the gyroscope and $\omega_{t}^{b}$ is the real angular rate. $b_{g}^{b}$ is the gyroscope bias in the ***b***-frame. $C_{gsc}$ and $C_{gno}$ are, respectively, the scale factor error matrix and non-orthogonal matrix of the gyroscope, $C_{gmis}$ is a misalignment error matrix between gyroscope and magnetometer, $C_{g}$ is a combination matrix of the above three, and $\varepsilon_{g}$ and $\varepsilon_{u}$ are independent zero-mean Gaussian white-noise.

In this paper, considering that the method is designed for low-cost MEMS navigation systems, a computationally expensive calibration process cannot be afforded. At the same time, to improve the observability of parameters, the scale factors and the non-orthogonal are considered as the calibrated parameters before leaving the factory. Therefore, only the gyroscope bias is considered and the gyroscope model is simplified as

(20) $$\begin{array}{c} \omega_{g}^{b}=\omega_{t}^{b}+b_{g}^{b}+\varepsilon_{g}\\ {\dot{b}}_{g}^{b}=\varepsilon_{u} \end{array}.$$

After establishing the gyroscope model, the relationship between the gyroscope bias and the magnetometer needs to be found. From Equation (9), we can obtain

(21) $$\left(I_{3\times 3}+C^{l}\right)B_{m}^{l}=C_{n}^{l}H^{n}+C_{b}^{l}b_{m}^{b}+\varepsilon_{m}.$$

The derivative of Equation (21) can then be shown by

(22) $$\left(I_{3\times 3}+C^{l}\right){\dot{B}}_{m}^{l}={\dot{C}}_{n}^{l}H^{n}+C_{n}^{l}{\dot{H}}^{n}+{\dot{C}}_{b}^{l}b_{m}^{b}+{\dot{\varepsilon }}_{m},$$

(23) $${\dot{C}}_{n}^{l}=-{\left[\omega_{g}^{b}\times \right]}_{n}^{l}C_{n}^{l},$$

(24) $${\dot{C}}_{b}^{l}=-{\left[\omega_{g}^{b}\times \right]}_{b}^{l}C_{b}^{l}.$$

As vehicles usually rotate more slowly than the sampling rate, ${\dot{C}}_{n}^{l}$ and ${\dot{C}}_{b}^{l}$ are expressed approximately in Equations (23) and (24). ${\left[\omega_{g}^{b}\times \right]}_{n}^{l}$ and ${\left[\omega_{g}^{b}\times \right]}_{b}^{l}$ are defined as follows:

(25) $${\left[\omega_{g}^{b}\times \right]}_{n}^{l}=\left[\begin{array}{ccc} 0 & -\omega_{gz}^{b} & 0\\ \omega_{gz}^{b} & 0 & 0\\ 0 & 0 & 0 \end{array}\right],$$

(26) $${\left[\omega_{g}^{b}\times \right]}_{b}^{l}=\left[\begin{array}{ccc} 0 & 0 & -\omega_{gy}^{b}\\ 0 & 0 & \omega_{gx}^{b}\\ \omega_{gy}^{b} & -\omega_{gx}^{b} & 0 \end{array}\right].$$

Combining Equations (14) and (22), we can obtain

(27) $$\begin{array}{cc} C_{n}^{l}{\dot{H}}^{n} & =\left(I_{3\times 3}+C^{l}\right){\dot{B}}_{m}^{l}-{\dot{C}}_{n}^{l}H^{n}-{\dot{C}}_{b}^{l}b_{m}^{b}-{\dot{\varepsilon }}_{m}\\ & =\left(I_{3\times 3}+C^{l}\right){\dot{B}}_{m}^{l}+{\left[\omega_{g}^{b}\times \right]}_{n}^{l}C_{n}^{l}H^{n}+{\left[\omega_{g}^{b}\times \right]}_{b}^{l}C_{b}^{l}b_{m}^{b}-{\dot{\varepsilon }}_{m}\\ & =\left(I_{3\times 3}+C^{l}\right){\dot{B}}_{m}^{l}+{\left[\omega_{g}^{b}\times \right]}_{n}^{l}\left(I_{3\times 3}+C^{l}\right)\left(B_{m}^{l}-C_{b}^{l}b_{m}^{b}\right)\\ & +{\left[\omega_{g}^{b}\times \right]}_{b}^{l}C_{b}^{l}b_{m}^{b}-{\left[\omega_{g}^{b}\times \right]}_{n}^{l}\varepsilon_{m}-{\dot{\varepsilon }}_{m} \end{array}$$

Some parameters are defined as follows:

(28) $$t_{1}=\left(I_{3\times 3}+C^{l}\right){\dot{B}}_{m}^{l},$$

(29) $$t_{2}={\left[\omega_{g}^{b}\times \right]}_{n}^{l}\left(I_{3\times 3}+C^{l}\right)\left(B_{m}^{l}-C_{b}^{l}b_{m}^{b}\right),$$

(30) $$t_{3}={\left[\omega_{g}^{b}\times \right]}_{b}^{l}C_{b}^{l}b_{m}^{b},$$

(31) $$t_{4}=t_{1}+t_{2}+t_{3}.$$

From Equations (27) to (31), the norm of ${\dot{H}}^{n}$ can be presented by

(32) $$\begin{array}{rr} \|{\dot{H}}^{n}{\|}^{2} & =\|t_{1}{\|}^{2}+\|t_{2}{\|}^{2}+\|t_{3}{\|}^{2}+2t_{1}^{T}t_{2}+2t_{1}^{T}t_{3}+2t_{2}^{T}t_{3}\\ & +{\left\|{\left[\omega_{g}^{b}\times \right]}_{n}^{l}\varepsilon_{m}\right\|}^{2}+{\left\|{\dot{\varepsilon }}_{m}\right\|}^{2}-2t_{4}^{T}{\left[\omega_{g}^{b}\times \right]}_{n}^{l}\varepsilon_{m}-2{\left(t_{4}-{\left[\omega_{g}^{b}\times \right]}_{n}^{l}\varepsilon_{m}\right)}^{T}{\dot{\varepsilon }}_{m} \end{array}.$$

Since ${\left\|{\dot{H}}^{n}\right\|}^{2}={\left\|{\dot{B}}_{m}^{l}\right\|}^{2}+{\left({\dot{B}}_{m}^{l}\right)}^{T}\left(2C^{l}+{\left(C^{l}\right)}^{2}\right){\dot{B}}_{m}^{l}$, the measuring equation of the MEMS gyroscope calibration model can be obtained as:

(33) $$\begin{array}{c} {\left\|{\dot{H}}^{n}\right\|}^{2}-{\left\|{\dot{B}}_{m}^{l}\right\|}^{2}={\left({\dot{B}}_{m}^{l}\right)}^{T}\left(2C^{l}+{\left(C^{l}\right)}^{2}\right){\dot{B}}_{m}^{l}+{\left\|t_{2}\right\|}^{2}+{\left\|t_{3}\right\|}^{2}\\ +2t_{1}^{T}t_{2}+2t_{1}^{T}t_{3}+2t_{2}^{T}t_{3}+w_{2}\\ w_{2}=\|{\left[\omega_{g}^{b}\times \right]}_{n}^{l}\varepsilon_{m}{\|}^{2}+\|{\dot{\varepsilon }}_{m}{\|}^{2}-2t_{4}^{T}{\left[\omega_{g}^{b}\times \right]}_{n}^{l}\varepsilon_{m}-2{\left(t_{4}-{\left[\omega_{g}^{b}\times \right]}_{n}^{l}\varepsilon_{m}\right)}^{T}{\dot{\varepsilon }}_{m} \end{array}.$$

### 2.3. Filter Algorithm

According to the above analysis, the magnetometer and gyroscope calibration problem can be formulated as a state estimation problem. Equations (16) and (33) are taken as the observation model and the state transition model is expressed as follows:

State transition model:

(34) $${\dot{C}}^{l}=0,$$

(35) $${\dot{b}}_{m}^{b}=0,$$

(36) $${\dot{b}}_{g}^{b}=0.$$

Observation model:

(37) $$\|H^{n}{\|}^{2}-\|B_{m}^{l}{\|}^{2}={\left(B_{m}^{l}\right)}^{T}\left(2C^{l}+{\left(C^{l}\right)}^{2}\right)B_{m}^{l}-2{\left(B_{m}^{l}\right)}^{T}\left(I_{3\times 3}+C^{l}\right)C_{b}^{l}b_{m}^{b}+\|b_{m}^{b}{\|}^{2},$$

(38) $$\|{\dot{H}}^{n}{\|}^{2}-\|{\dot{B}}_{m}^{l}{\|}^{2}={\left({\dot{B}}_{m}^{l}\right)}^{T}\left(2C^{l}+{\left(C^{l}\right)}^{2}\right){\dot{B}}_{m}^{l}+\|t_{2}{\|}^{2}+\|t_{3}{\|}^{2}+2t_{1}^{T}t_{2}+2t_{1}^{T}t_{3}+2t_{2}^{T}t_{3}.$$

In the above, the calibration parameters of the magnetometer and the gyroscope are constant, such that the rate of change of these parameters is equal to zero in the state transition model. For the observation model, it is known that the calibration system is a nonlinear system and the Jacobian matrix of the observation model is difficult to obtain. Hence, the appropriate filter should be employed for rapid and precise calibration. In this paper, CKF is applied for magnetometer and gyroscope calibration since it has some advantages, including fast convergence and less computational needs [28].

CKF is a filter method based on the cubature transform. The core of CKF is the cubature transformation by a spherical-radial rule [29]. It takes advantage of a set of cubature points to propagate state and covariance matrices of the system. The time update process of the CKF algorithm is expressed as:

(39) $$S_{k-1/k-1}=SVD\left(P_{k-1/k-1}\right),$$

(40) $$\chi_{k-1/k-1}=S_{k-1/k-1}\xi +x_{k-1/k-1},$$

(41) $$\chi_{k/k-1}^{*}=f\left(\chi_{k-1/k-1}\right),$$

(42) $$x_{k/k-1}=\frac{1}{m}\sum_{i=1}^{m}\chi_{i,k/k-1}^{*},$$

(43) $$P_{k/k-1}=\frac{1}{m}\sum_{i=1}^{m}\chi_{i,k/k-1}^{*}\chi_{i,k/k-1}^{*T}-x_{k/k-1}x_{k/k-1}^{T}+Q_{k},$$

where SVD(·) represents the singular value decomposition of the matrix and f(·) is the state transition function. Matrix $S_{k-1/k-1}$ is the root mean square of the covariance matrix P, m = 2n (n is the dimensions of the system), $\xi =\sqrt{m/2}{\left[1\right]}_{i}$ (For example, when n = 2, [1] = {(1,0)^T^,(−1,0)^T^,(0,1)^T^,(0,−1)^T^}, [1] *_i_* represents the *i*th column of set [1]). However, because the state vector of the calibration system is constant, the state transfer matrix is a unit matrix, which allows the state transition errors to be ignored; in other words, $Q_{k}$ is a zero matrix. The time update can be simplified as follows:

(44) $$P_{k/k-1}\approx P_{k-1/k-1},$$

(45) $$x_{k/k-1}\approx x_{k-1/k-1}.$$

This solution simplifies the operation of updating time, which also reduces computational needs. The CKF measurement update process is summarized as follows:

(46) $$S_{k/k-1}=SVD\left(P_{k/k-1}\right),$$

(47) $$\chi_{k/k-1}=S_{k/k-1}\xi +x_{k/k-1},$$

(48) $$V_{k/k-1}=h\left(\chi_{k/k-1}\right),$$

(49) $$v_{k/k-1}=\frac{1}{m}\sum_{i=1}^{m}V_{i,k/k-1},$$

(50) $$P_{zz,k/k-1}=\frac{1}{m}\sum_{i=1}^{m}V_{i,k/k-1}V_{i,k/k-1}^{T}-v_{k/k-1}v_{k/k-1}^{T}+R_{k},$$

(51) $$P_{xz,k/k-1}=\frac{1}{m}\sum_{i=1}^{m}\chi_{k/k-1}V_{i,k/k-1}^{T}-x_{k/k-1}v_{k/k-1}^{T},$$

(52) $$K_{k}=P_{xz,k/k-1}P_{zz,k/k-1}^{-1},$$

(53) $$x_{k/k}=x_{k-1/k-1}+K_{k}\left(z_{k}-v_{k/k-1}\right),$$

(54) $$P_{k/k}=P_{k/k-1}-K_{k}P_{zz,k/k-1}K_{k}^{T},$$

where h(·) is the observation function. $x_{k/k}$ and $P_{k/k}$ are the estimated results at step *k*, and represent the state vector and state covariance matrix separately.

## 3. Simulation Test

In this section, the feasibility of the proposed method will be verified by simulation testing. In the following, the simulation details will be introduced.

### 3.1. Reference Algorithm

The proposed method aims to solve the problem that existing calibrations methods do not consider the situation that only level rotation is performed. Existing calibration methods need data in all possible directions and cannot be used with only level rotation. To check the effect of the proposed method, the normal attitude-independent method (NAIM) [8,9] and the magnetometer and gyroscope integrated calibration method (MGICM) are compared in the specified rotation and simulation parameters [21,22]. The NAIM is a classic magnetometer calibration method without any attitude information. This method uses an attitude-independent model for magnetometer calibration by removing the attitude matrix, and CKF is used to estimate calibration parameters. In this algorithm, according to Equation (5), the magnetometer calibration model is set as

(55) $$B_{m}^{b}={\left(I_{3\times 3}+C^{b}\right)}^{-1}B_{t}^{b}+b_{m}^{b}+\varepsilon_{m},$$

(56) $$C^{b}=\left[\begin{array}{ccc} C_{11}^{b} & C_{12}^{b} & C_{13}^{b}\\ C_{12}^{b} & C_{22}^{b} & C_{23}^{b}\\ C_{13}^{b} & C_{23}^{b} & C_{33}^{b} \end{array}\right],$$

in which the scale factor matrix $C^{b}$ is symmetric. $B_{m}^{b}$ and $b_{m}^{b}$ are located in the ***b***-frame and the attitude matrix $C_{n}^{b}$ is removed by the normalized process of Equation (55). Then, the gyroscope calibration model is deduced. The NAIM calibration system is expressed as follows:

State transition model:

(57) $${\dot{C}}^{b}=0,$$

(58) $${\dot{b}}_{m}^{b}=0,$$

(59) $${\dot{b}}_{g}^{b}=0.$$

Observation model:

(60) $$\|H^{n}{\|}^{2}-\|B_{m}^{b}{\|}^{2}={\left(B_{m}^{b}\right)}^{T}\left(2C^{b}+{\left(C^{b}\right)}^{2}\right)B_{m}^{b}-2{\left(B_{m}^{b}\right)}^{T}\left(I_{3\times 3}+C^{b}\right)b_{m}^{b}+\|b_{m}^{b}{\|}^{2},$$

(61) $$\|{\dot{H}}^{n}{\|}^{2}-\|{\dot{B}}_{m}^{b}{\|}^{2}={\left({\dot{B}}_{m}^{b}\right)}^{T}\left(2C^{b}+{\left(C^{b}\right)}^{2}\right){\dot{B}}_{m}^{b}+\|t_{2}{\|}^{2}+2t_{1}^{T}t_{2},$$

in which

(62) $$t_{1}=\left(I_{3\times 3}+C^{b}\right){\dot{B}}_{m}^{b},$$

(63) $$t_{2}={\left[\omega_{g}^{b}\times \right]}_{n}^{b}\left(I_{3\times 3}+C^{b}\right)\left(B_{m}^{b}-b_{m}^{b}\right),$$

(64) $${\left[\omega_{g}^{b}\times \right]}_{n}^{b}=\left[\begin{array}{ccc} 0 & -\omega_{gz}^{b} & \omega_{gy}^{b}\\ \omega_{gz}^{b} & 0 & -\omega_{gx}^{b}\\ -\omega_{gy}^{b} & \omega_{gx}^{b} & 0 \end{array}\right].$$

The MGICM calibrates the magnetometer using the angular velocity from the gyroscope, while gyroscope calibration parameters are simultaneously estimated using the calibrated magnetometer readings. The state transition model and observation model are summarized as follows:

State transition model:

(65) $${\dot{C}}_{b}^{n}=C_{b}^{n}\left(\omega_{g}^{b}+b_{g}^{b}\right)\times ,$$

(66) $${\dot{C}}^{b}=0,$$

(67) $${\dot{b}}_{m}^{b}=0,$$

(68) $${\dot{b}}_{g}^{b}=0.$$

Observation model:

(69) $$B_{m}^{b}={\left(I_{3\times 3}+C^{b}\right)}^{-1}C_{n}^{b}H^{n}+b_{m}^{b}.$$

To guarantee fairness, this algorithm will use CKF to estimate parameters.

### 3.2. Simulation Setting

**Figure 2:**
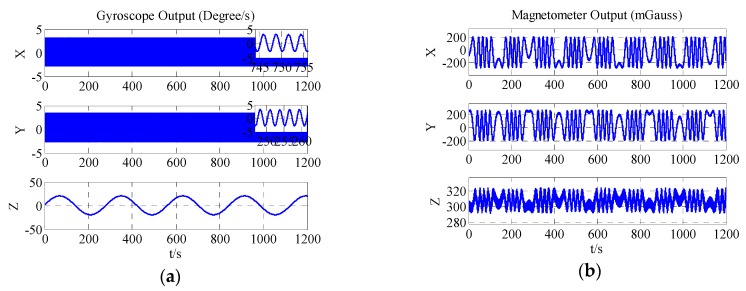

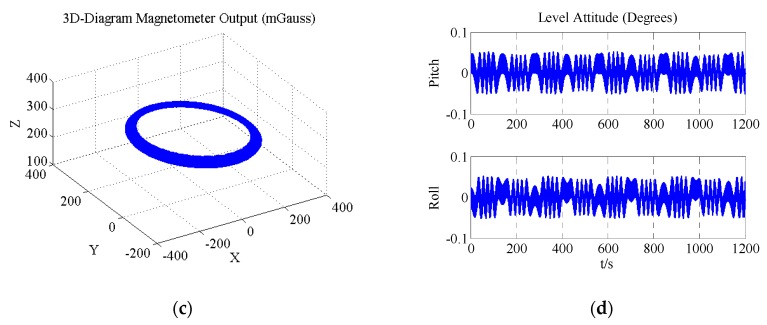
(**a**) Three-axis gyroscope output; (**b**) Three-axis magnetometer output; (**c**) 3D-diagram magnetometer output; (**d**) Level attitude of system.

The proposed method is considered for the situation that the navigation system on many vehicles cannot rotate around two or more axes, such that traditional magnetometer and gyroscope calibration methods may give the wrong results. To check the effectiveness of the proposed method, the rotation model is set to level rotation, where the system rotates only around the *z*-axis and a limited angular rate may exist on the other two axes. Table 2 provides simulation settings. More detailed rotations are shown in Figure 2. Figure 2a shows the gyroscope output and Figure 2b demonstrates the three-axis magnetometer output. The three-dimensional diagram of magnetometer output is displayed in Figure 2c. The system level attitude is shown in Figure 2d. From Figure 2c, it is clearly observed that the locus of magnetometer data is an approximate ellipse rather than ellipsoid.

The proposed method needs the level attitude of the system to assist calibration. The level attitude can be obtained by any one of the following methods [8]:

1. The level attitude can be gained through accelerometer measures of the orientation of gravity. This method has high precision and stability when static, but cannot guarantee those advantages in dynamic conditions.
2. The gyroscope can update a known attitude by several methods, including quaternions. The advantage is high precision and stability over a short period of time. It is prone to accumulate errors over longer time periods.
3. An integrated navigation system can provide the precision attitude by integrating different sensors with integration algorithms such as KF and EKF, and so on. These sensors may include a gyroscope, accelerometer, odometer, GNSS, Wi-Fi, etc. This method can guarantee high precision and stability over a long time period, and can be applied in any environment if a suitable integration algorithm is chosen.

### 3.3. Simulation Result

**Figure 3:**
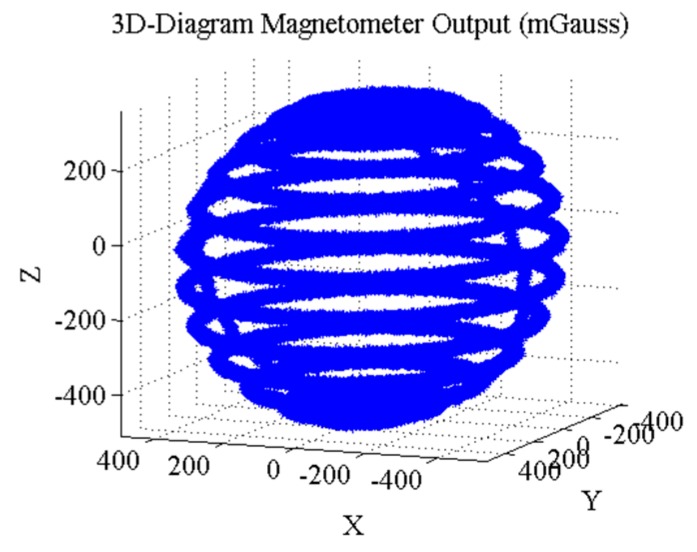
3D diagram of data in all possible directions.

First, to verify the effectiveness of NAIM and MGICM, the data in all possible directions are used to achieve calibration. Figure 3 shows the 3D diagram of data in all possible directions.

**Figure 4:**
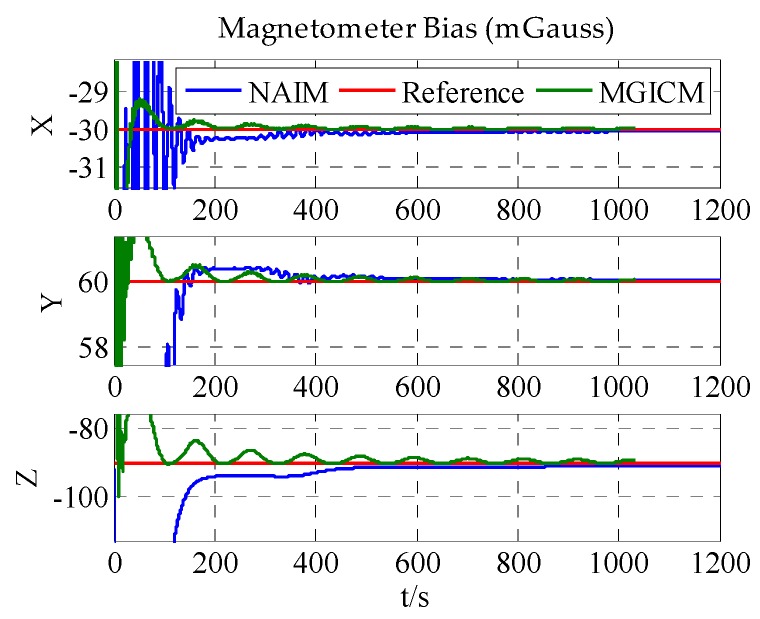
Estimation result of magnetometer bias. MGICM: magnetometer and gyroscope integrated calibration method; NAIM: normal attitude-independent method.

**Figure 5:**
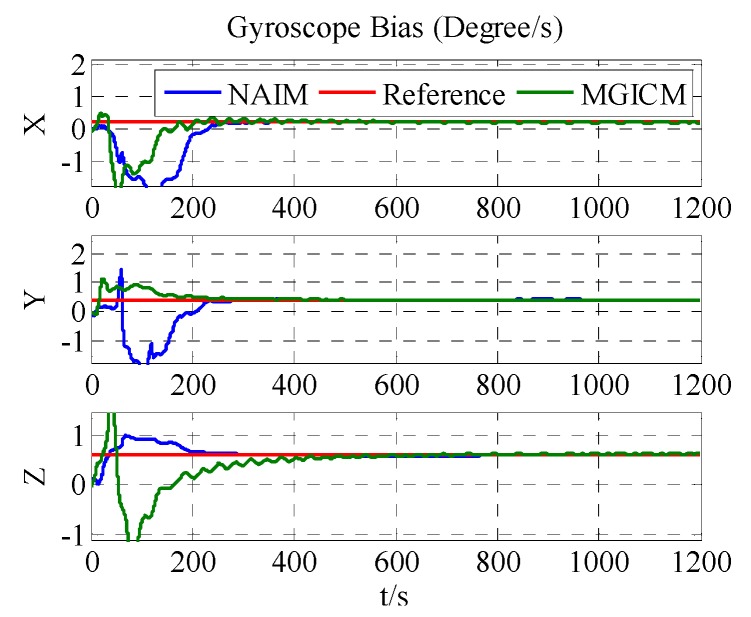
Estimation result of gyroscope bias.

**Figure 6:**
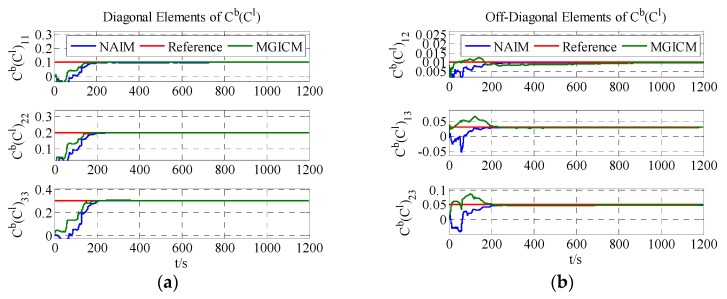
Estimation result of magnetometer scale factors.

**Figure 7:**
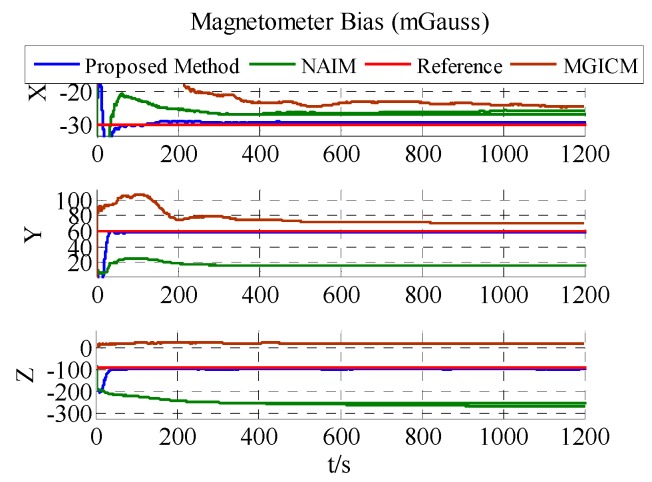
Estimation result of magnetometer bias.

**Figure 8:**
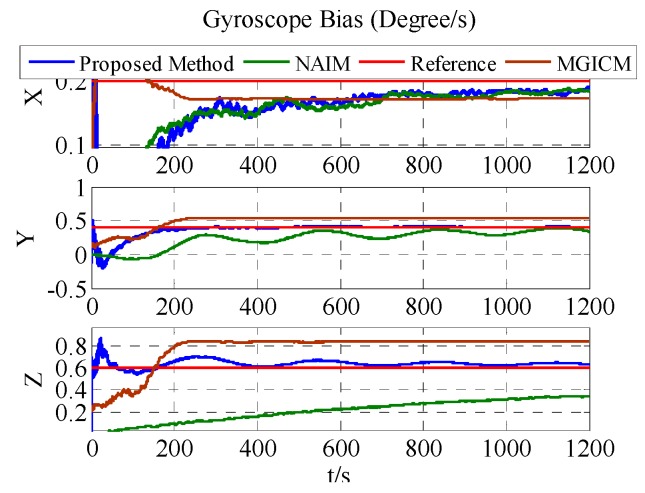
Estimation result of gyroscope bias.

**Figure 9:**
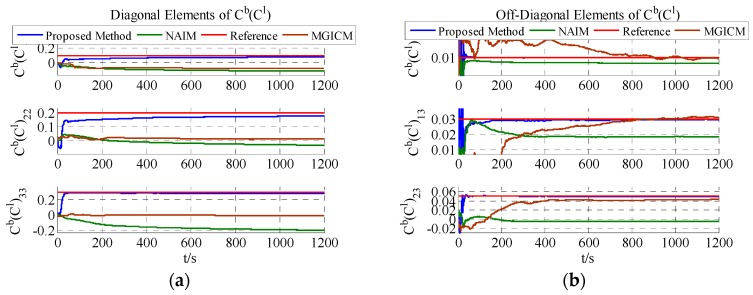
Estimation result of magnetometer scale factors.

The calibration results are summarized in Figure 4, Figure 5 and Figure 6. As shown in Figure 4, Figure 5 and Figure 6, a magnetometer and gyroscope can be calibrated accurately. In other words, the two reference algorithms are effective. Then, without changing any parameters, the level rotation data is used to achieve calibration. With level rotation, the simulation results are as shown in Figure 7, Figure 8 and Figure 9.

The calibration results are summarized in Table 3. In Figure 7, Figure 8 and Figure 9, the red line is the reference value, and the blue, green, and brown lines represent the estimation results of the proposed method, NAIM, and MGICM. Figure 7 and Figure 9 show the estimation result of the magnetometer, from which it is evident that the results of the NAIM and MGICM converge to incorrect values; by contrast, the results of the proposed method gradually converge to the reference. The result of magnetometer calibration is the basis of gyroscope calibration. Figure 8 indicates the simulation results of gyroscope bias. In Figure 8, the estimation results of the proposed method quickly and accurately converge to the reference values. In comparison, the estimation of NAIM and MGICM cannot get close to the reference. In conclusion, when the system only rotates around the *z*-axis, the NAIM and MGICM cannot calibrate the system because of a lack of sufficient data in that direction. However, the proposed method can complete calibration successfully, which indicates that it boasts better adaptability and important application value in actual systems.

## 4. Experimental Test

To verify the practical application of the above algorithm, this section will take advantage of an actual MEMS-IMU integrated system to verify the effect of the algorithm.

### 4.1. Test Condition

**Figure 10:**
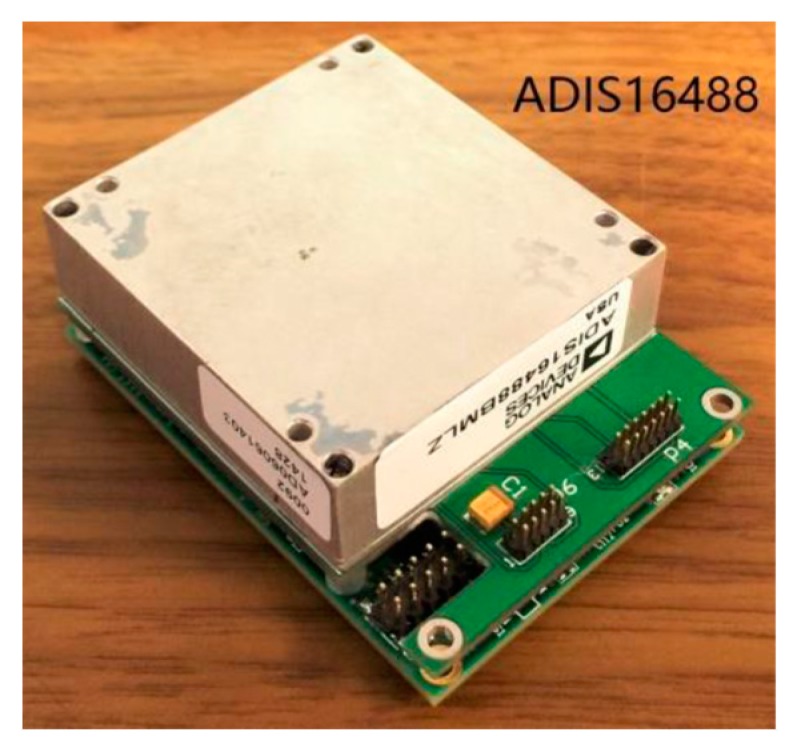
ADIS16488 micro-electro-mechanical-system inertial measurement unit (MEMS-IMU) system.

This test was performed indoors and in a stable magnetic field environment. In the test, we chose ADIS16488 as the MEMS-IMU integrated system, as indicated in Figure 10. The features of ADIS16488 are shown in Table 4. ADIS16488 rotated around the *z*-axis with a certain angular velocity by hand and the gyroscope and magnetometer data were collected simultaneously.

**Figure 11:**
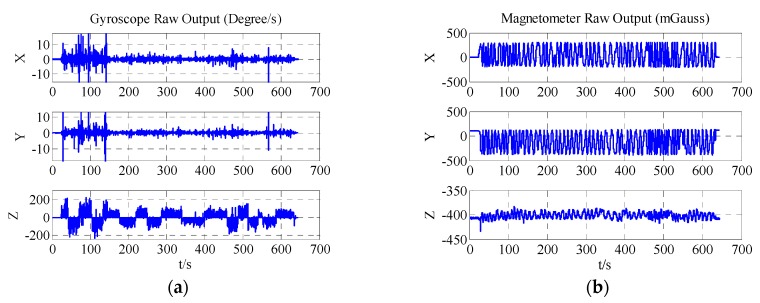

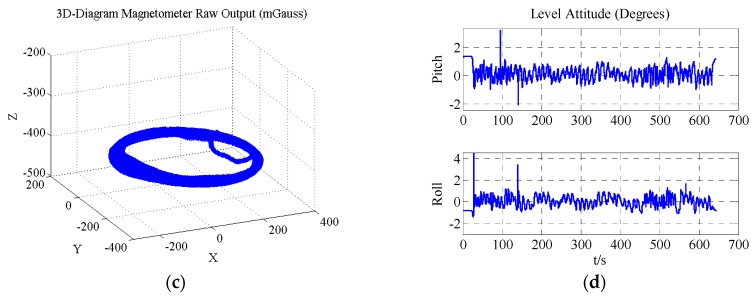
(**a**) Output of the ADIS16488 gyroscope; (**b**) Output of the ADIS16488 magnetometer; (**c**) Three-dimensional diagram of ADIS16488′s magnetometer output; (**d**) Level attitude of the system.

Panels a and b of Figure 11 display the three-axis raw output of ADIS16488′s gyroscope and magnetometer. Figure 11c demonstrates the three-dimensional diagram of ADIS16488′s magnetometer output. Figure 11d shows the level attitude of the system. This level attitude was calculated by the accelerometer, and the calculation method is provided in Section 3.2.

### 4.2. Test Results

**Figure 12:**
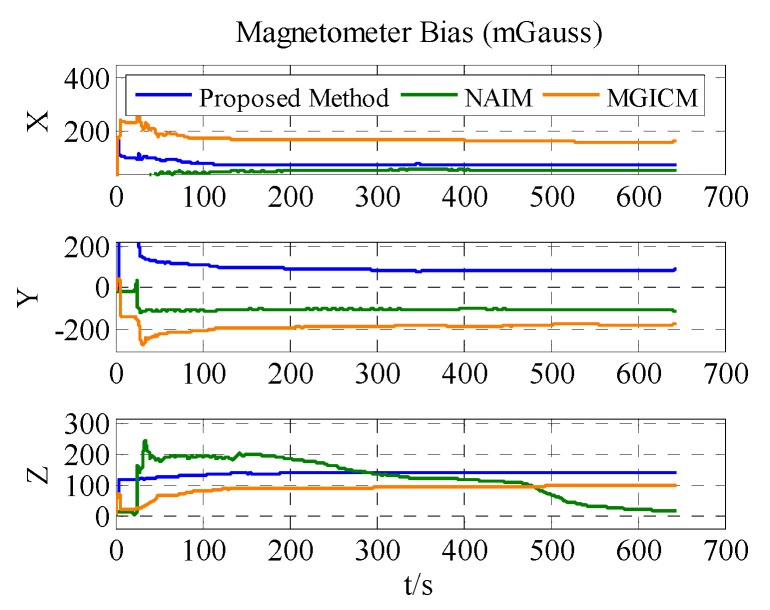
Estimation results of ADIS16488′s magnetometer bias.

**Figure 13:**
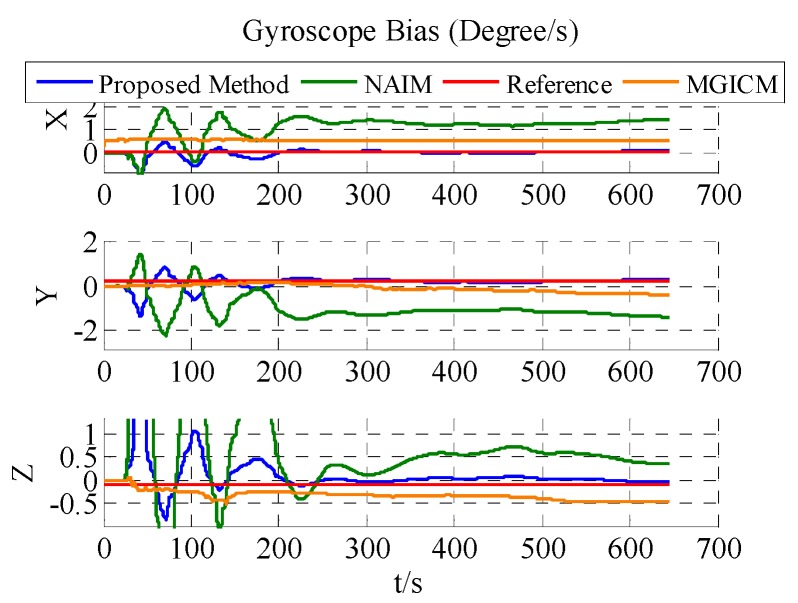
Estimation result of ADIS16488′s gyroscope bias.

**Figure 14:**
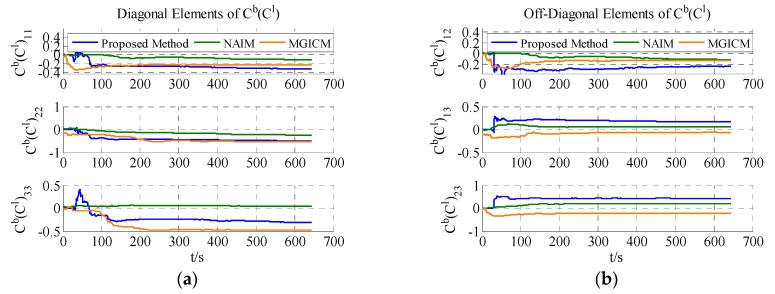
Estimation results of ADIS16488′s magnetometer scale factors.

The results of the actual experiment are listed in Table 5. In Figure 12, Figure 13 and Figure 14, the red line expresses the reference. The blue, green, and brown lines show the results of the proposed method, NAIM, and MGICM, respectively. Due to the reference values of the magnetometer, parameters are difficult to know, so gyroscope calibration results could be used to verify magnetometer calibration results. From the velocity of convergence, the calibration of the magnetometer was completed first, and then the gyroscope was gradually calibrated. In other words, if the gyroscope is calibrated accurately, the magnetometer is also calibrated precisely. To check the gyroscope calibration results, the approximate bias of the gyroscope can be obtained by letting the system remain stable for a long time. Therefore, it will be applied to verify the calibration results as the reference values. In Figure 13, it is obvious that the result of the proposed method only gets close to the reference and the other results are wrong, which indicates that the proposed method is correct and valid. Moreover, the calibration of the magnetometer can be completed within 200 s, and the calibration of the gyroscope bias is finished in about 600 s. From the above results, the proposed method can be used accurately with level rotation, otherwise the general calibration model may obtain an incorrect result when using only level rotation.

## 5. Conclusions

Aiming at an actual problem (i.e., that most calibration methods require that the sensors be rotated in all possible orientations to guarantee a good result), it is, however, impossible for the navigation system on vehicles such as cars, ships, or planes. Therefore, a gyroscope and magnetometer integrated with an on-line calibration method is proposed with level rotation. This work mainly provides two contributions: (1) Relying on the level attitude, the magnetometer output model is redefined and the level two-dimensional gyroscope and magnetometer calibration model is deduced. (2) A simple CKF is designed to rapidly and accurately complete calibration. Then, the NAIM and MGICM are introduced to check the simulated effect of the proposed method. With level rotation, the NAIM and MGICM obtain the wrong calibration results, while the calibration result of the proposed method is close to the reference. In the actual test, an ADIS16488 MEMS-IMU integrated system is used to verify the practicability of the proposed method. The test data were obtained by rotating the system around the *z*-axis with a certain angular velocity by hand. Finally, the proposed method displays good effects, which illustrates that the method possesses strong practicability.

## Figures and Tables

**Table 1:** Comparison of magnetometer calibration methods.

Method	Inputs	Advantages	Disadvantages
Ellipsoid fitting [4,5]	Magnetic field	Simple, Short run time, Less calculation	Low precision, Bad adaptability, Off-line, Rotation in all possible orientations
Attitude-dependent [6,7]	Magnetic field, Heading	Data in small space coverage, Model precision, Short run time	Calibration precision, Relies on heading
Attitude-independent [8,9,10,11,12,13]	Magnetic field	Model precision, On-line, Good adaptability	Hard to set initial filter parameter, Rotation in all possible orientations
Particle swarm optimization algorithm [18,19,20]	Magnetic field	Need not set initial parameters, Model precision	High calculation cost, Rotation in all possible orientations, Off-line
Gyroscope integrated calibration [21,22]	Magnetic field, Angular rates	Model precision, On-line	Rotation in all possible orientations
Virtual rotation scheme [23,24,25]	Angular rates	Model precision, Does not require initial situation	High calculation cost, Has not been used to calibrate magnetometer
Proposed method	Magnetic field, Angular rates, Level attitude	On-line, Only needs level rotation	Relatively obscure model, Relies on level attitude

**Table 2:** Simulation setting.

Items	Values
Device rotation angular rate on *z*-axis	Less than 20 (degree/s) in sine wave with 300 s periods
Device rotation angular rate on x and y-axis	Less than 3 (degree/s) in sine wave with 3 s periods
Update frequency	200 (Hz)
$b_{m}^{b}$	[−30,60,90]^T^ (mGauss)
$C^{l}$ (Proposed method) $C^{b}$ (Attitude-independent method)	$\left[\begin{array}{ccc} 0.1 & 0.01 & 0.03\\ 0.01 & 0.2 & 0.05\\ 0.03 & 0.05 & 0.3 \end{array}\right]$
$b_{g}^{b}$	[0.2,0.3,0.4]^T^ (degree/s)
$\varepsilon_{g}$	0.2 (degree/s)(rms)
$\varepsilon_{m}$	0.45 (mGauss) (rms)

**Table 3:** Simulation results.

Method	$b^{b}$	$C^{b}\left(C^{l}\right)$	$b_{g}^{b}$
Reference	$\left[\begin{array}{c} -30\\ 60\\ -90 \end{array}\right]$ (mGauss)	$\left[\begin{array}{ccc} 0.1 & 0.01 & 0.03\\ 0.01 & 0.2 & 0.05\\ 0.03 & 0.05 & 0.3 \end{array}\right]$	$\left[\begin{array}{c} 0.2\\ 0.4\\ 0.6 \end{array}\right]$ (degree/s)
NAIM	$\left[\begin{array}{c} -25.79\\ 15.95\\ -268.4 \end{array}\right]$ (mGauss)	$\left[\begin{array}{ccc} -0.112 & -0.007 & 0.018\\ -0.007 & -0.034 & -0.004\\ 0.018 & -0.004 & -0.198 \end{array}\right]$	$\left[\begin{array}{c} 0.1865\\ 0.3427\\ 0.338 \end{array}\right]$ (degree/s)
MGICM	$\left[\begin{array}{c} -24.39\\ 69.68\\ 21.54 \end{array}\right]$ (mGauss)	$\left[\begin{array}{ccc} -0.0737 & 0.0094 & 0.0315\\ 0.0094 & 0.0102 & 0.0432\\ 0.0315 & 0.0432 & -0.004 \end{array}\right]$	$\left[\begin{array}{c} 0.1732\\ 0.5313\\ 0.8376 \end{array}\right]$ (degree/s)
Proposed method	$\left[\begin{array}{c} -29.24\\ 58.74\\ -96.79 \end{array}\right]$ (mGauss)	$\left[\begin{array}{ccc} 0.08 & 0.009 & 0.029\\ 0.009 & 0.178 & 0.049\\ 0.029 & 0.049 & 0.285 \end{array}\right]$	$\left[\begin{array}{c} 0.19\\ 0.406\\ 0.6303 \end{array}\right]$ (degree/s)

**Table 4:** ADIS16488’s features.

Items	ADIS16488
Sampling rates	205 (Hz)
Gyroscope bias repeatability	±0.2 (degree/s)
Gyroscope in-run bias stability	6.25 (degree/h)
Gyroscope angular random walk	0.3 (degree/√h)
Gyroscope output noise	0.16 (degree/s)(rms)
Accelerometer bias repeatability	±16 (mg)
Accelerometer in-run bias stability	0.1 mg
Accelerometer velocity random walk	0.029 m/s/√h
Accelerometer output noise	1.5 (mg)(rms)
Magnetometer output noise	0.45 (mGauss)(rms)

**Table 5:** Calibration results.

Method	$b^{b}$	$C^{b}\left(C^{l}\right)$	$b_{g}^{b}$
Reference	-	-	(degree/s)
NAIM	$\left[\begin{array}{c} 161.8\\ 111\\ 18.53 \end{array}\right]$ (mGauss)	$\left[\begin{array}{ccc} -0.112 & -0.109 & 0.58\\ -0.109 & -0.034 & 0.188\\ 0.058 & 0.188 & -0.198 \end{array}\right]$	$\left[\begin{array}{c} 1.431\\ -1.396\\ 0.345 \end{array}\right]$ (degree/s)
MGICM	$\left[\begin{array}{c} 51.42\\ 178.9\\ 98.64 \end{array}\right]$ (mGauss)	$\left[\begin{array}{ccc} -0.293 & -0.127 & -0.064\\ -0.127 & -0.543 & -0.223\\ -0.064 & -0.223 & -0.485 \end{array}\right]$	$\left[\begin{array}{c} 0.5216\\ -0.39\\ -0.456 \end{array}\right]$ (degree/s)
Proposed method	$\left[\begin{array}{c} 74.27\\ 87\\ 140.2 \end{array}\right]$ (mGauss)	$\left[\begin{array}{ccc} -0.3136 & -0.2374 & 0.1702\\ -0.2374 & -0.4998 & 0.4287\\ 0.1702 & 0.4287 & -0.3118 \end{array}\right]$	$\left[\begin{array}{c} 0.074\\ 0.27\\ -0.0957 \end{array}\right]$ (degree/s)
